# Notch1 Inhibits Rosiglitazone-Induced Adipogenic Differentiation in Primary Thymic Stromal Cells

**DOI:** 10.3389/fphar.2018.01284

**Published:** 2018-11-12

**Authors:** Yajun Wang, Jianxin Tan, Hongmei Du, Xue Liu, Siliang Wang, Simeng Wu, Zhe Yuan, Xike Zhu

**Affiliations:** ^1^Research Center, Shengjing Hospital of China Medical University, Shenyang, China; ^2^State Key Laboratory of Reproductive Medicine, Department of Prenatal Diagnosis, The Affiliated Obstetrics and Gynecology Hospital of Nanjing Medical University, Nanjing Maternity and Child Health Care Hospital, Nanjing, China; ^3^Department of Medical Oncology, Shengjing Hospital of China Medical University, Shenyang, China; ^4^Department of Blood Transfusion, Shengjing Hospital of China Medical University, Shenyang, China

**Keywords:** thymic adipogenesis, thymic stromal cells, RNA-seq, Notch1, autophagy

## Abstract

Adipocyte deposition is believed to be a primary characteristic of age-related thymic involution. Herein, we cultured primary thymic stromal cells (TSCs), used rosiglitazone, a potent peroxisome proliferator-activated receptor γ (PPARγ) agonist, to induce adipogenic differentiation, and investigated the differentially expressed genes during adipogenic differentiation by using RNA-sequencing analysis. Furthermore, the effects of Notch1 on rosiglitazone-induced adipogenic differentiation of TSCs as well as the underlying mechanisms were also investigated. As a result, we identified a total of 1737 differentially expressed genes, among which 965 genes were up-regulated and 772 genes were down-regulated in rosiglitazone-treated cells compared with control cells. Gene ontology (GO) enrichment analysis showed that the GO terms were enriched in metabolic process, intracellular, and protein binding. Kyoto encyclopedia of genes and genomes (KEGG) analysis showed that a number of pathways, including ubiquitin mediated proteolysis, PPAR signaling pathway, and mammalian target of rapamycin (mTOR) signaling pathway were predominantly over-represented. Meanwhile, overexpression of Notch1 suppressed and inhibition of Notch1 promoted rosiglitazone-induced adipogenic differentiation in TSCs, and the pro-adipogenic effects of the Notch inhibitor DAPT were associated with the activation of autophagy. Taken together, our results suggest that Notch1 is a key regulator in thymic adipogenesis and may serve as a potential target to hinder thymic adiposity in age-related thymic involution.

## Introduction

The thymus is a unique primary lymphoid organ, which provides the inductive microenvironments to ensure the differentiation, development, and maturation of T cells (Chaudhry et al., 2016). Unlike most other organs, the thymus enlarges from infancy to adolescence but begins to slowly shrink from puberty onwards (Aw and Palmer, 2011). Termed as age-related involution, this physiological process is characterized by decrease in thymic size, abnormal thymic architecture, and the replacement of thymic epithelial cells with adipocytes, eventually leading to decrease in naïve T cell output and increased risk to infection, autoimmune disease, and cancer in the elderly (Gui et al., 2012). Adipocyte deposition is believed to be a primary characteristic of age-related thymic involution and contributes to the deterioration of the thymic microenvironments (Flores et al., 1999; Ventevogel and Sempowski, 2013). Recent evidence suggests that the transdifferentiation of thymic stromal cells (TSCs) into adipocytes is a possible source of thymic adipogenesis (Youm et al., 2009; Tan J. et al., 2016). However, the underlying mechanisms involved in thymic adiposity remain to be clarified.

The next generation sequencing technology, a state-of-the-art, large-scale and systematic analysis, has been widely used in transcriptomic studies, termed as RNA-sequencing (RNA-seq), which enable us to simultaneously characterize 1000s of transcripts expressed in defined biological samples (Wang et al., 2009; McGettigan, 2013). Because of its advantages in high throughput and low cost, RNA-seq is rapidly replacing the microarray technology as the first choice for gene expression studies (Zhao et al., 2014). To the best of our knowledge, however, there have been no reports using RNA-seq analysis to identify key regulators in thymic adipogenesis.

Peroxisome proliferator-activated receptor γ (PPARγ) is a key player in the differentiation of adipocytes, and constitutive PPARγ activation induces ectopic adipogenesis and promotes age-related thymic involution in mice (Youm et al., 2010; Ernszt et al., 2017). Furthermore, rosiglitazone, a potent PPARγ agonist, has been successfully used to induce adipogenic differentiation of OP9-DL1 cells and primary TSCs *in vitro* (Yang et al., 2009; Tan et al., 2017). In order to identify key regulators in thymic adipogenesis, we cultured primary TSCs, used rosiglitazone to induce adipogenic differentiation, and investigated the differentially expressed genes during adipogenic differentiation by using RNA-seq analysis. Furthermore, we found that Notch signaling pathway is involved in adipogenic differentiation of TSCs. Finally, we evaluated whether Notch1 inhibits rosiglitazone-induced adipogenic differentiation in TSCs and, if so, whether the effects of Notch1 are associated with the regulation of autophagy in TSCs.

## Materials and Methods

### Animals and Chemicals

C57BL/6 mice at ages of 2–3 months were obtained from Huafukang Bioscience Co. Ltd. (Beijing, China) and housed under specific pathogen-free conditions in the animal care facility at Shengjing Hospital of China Medical University. All animal experiment protocols were reviewed and approved by the Animal Ethics Committee of Shengjing Hospital. The Notch inhibitor DAPT (N-[N-(3, 5-Difluorophenacetyl)-L-alanyl]-S-phenylglycine t-butyl ester) and autophagy inhibitor 3-methyladenine (3-MA) were purchased from MedChem Express (Monmouth Junction, NJ, United States).

### Cell Culture

Primary TSCs were cultured from postnatal thymi by enzymatic digestion. Briefly, freshly dissected thymic lobes were cut into small fragments and washed with RPMI-1640 medium to remove the majority of thymocytes. Then, the thymic fragments were incubated for 20 min at 37°C with RPMI-1640 medium containing 0.05% Liberase^TH^ (Roche, Basel, Switzerland) and 200 U/ml DNase I (Sangon Biotechnology, Shanghai, China). Gentle mechanical agitation was performed, and the cell suspensions were collected and added into phosphate buffered saline (PBS) containing 0.5% bovine serum albumin (BSA) and 2 mM ethylenediaminetetraacetic acid (EDTA) to neutralize digestion. The remaining aggregates were further treated with enzymatic mixture, and the digestion was repeated thrice until completely dispersed. The cell suspensions were pooled, centrifuged, and resuspended in PBS solution. After filtered through 100 μm mesh, the cells were seeded into 24-well plate at a density of 10^6^/well and cultured in DMEM/F12 medium supplemented with 3 μg/ml insulin (Biovision, Livingston, NJ, United States), 20 ng/ml epidermal growth factor (Peprotech, Rocky Hill, NJ, United States), 100 U/ml penicillin, 100 g/ml streptomycin, and 20% fetal bovine serum (FBS; Biological Industries, Kibbutz Beit-Haemek, Israel) at 37°C in a 5% CO_2_ incubator (Yang et al., 2009). After attachment, the thymocytes were removed by refreshing the culture medium. To induce adipogenic differentiation, TSCs at passage 3 were incubated with 10 μM rosiglitazone (Aladdin, Shanghai, China) for 7 days, and the medium containing rosiglitazone was refreshed every other day. Dimethyl sulphoxide (DMSO)-treated cells were used as a vehicle control (Tan et al., 2017).

### RNA Extraction, Library Preparation for RNA-Seq

Total RNA was isolated by using RNAiso Plus (TAKARA, Dalian, China). The purity of RNA was assessed with a NanoPhotometer^®^ spectrophotometer (IMPLEN, Westlake Village, CA, United States). RNA concentrations were measured by using Qubit^®^ RNA Assay Kit on a Qubit 2.0 Flurometer (Life Technologies, Carlsbad, CA, United States), and RNA integrity was evaluated by using RNA Nano 6000 Assay Kit on the Bioanalyzer 2100 system (Agilent Technologies). For library preparation, rRNA was depleted by using the EpicentreRibo-zero^TM^ rRNA Removal Kit (Madison, WI, United States). Subsequently, fragmentation was performed in NEBNext first strand synthesis reaction buffer, and the first-strand cDNA was synthesized with a random hexamer primer and M-MuLV reverse transcriptase. DNA polymerase I and RNase H were used to synthesize the second-strand cDNA. After adenylation of the 3′ ends of the DNA fragment, the NEBNext adaptor was ligated, and 150–200 bp cDNA fragments were purified. PCR amplification was carried out to enrich the cDNA libraries using Phusion high-fidelity DNA polymerase, universal PCR primers, and index primer. The quality of library was assessed with the Agilent bioanalyzer 2100 system. The libraries were sequenced on an Illumina HiSeq 4000 platform (Illumina, San Diego, CA, United States), and 150 bp paired-end reads were obtained after clustering of the index-coded samples.

### Analysis of RNA-Seq Reads and Differentially Expressed Transcripts

Raw data in the Fastq format were analyzed by in-house perl scripts to obtain clean reads. The clean pair-end reads were aligned to the reference genome (hg38) using HISAT2, and the transcripts were merged by StringTie and Cuffmerge. Differential expression analysis was performed using the edgeR. Genes with *P* < 0.05 and fold changes > 2 were considered to be significantly differential expressed. KOBAS software was used to evaluate the statistical enrichment kyoto encyclopedia of genes and genomes (KEGG) pathways of differentially expressed genes.

### Real-Time PCR

Total RNA was isolated by using RNAiso Plus (TAKARA) following the instructions according to the manufacturers. Real-time PCR reactions were conducted using SYBR^®^ Premix Ex Taq^TM^ II (TAKARA) on the 7500 Fast Real-Time PCR System (Applied Biosystems, Foster City, CA, United States) as previously described (Tan et al., 2017). The primer sequences are shown in Table 1. The relative mRNA levels were calculated by the comparative threshold method using glyceraldehyde-3-phosphate dehydrogenase (GAPDH) as an internal control for normalization.

**Table 1:** Sequences of primers used for real-time PCR.

Gene symbol		Sequence (5′–3′)
PPARγ	Forward	TTTTCCGAAGAACCATCCGATT
	Reverse	ATGGCATTGTGAGACATCCCC
FABP4	Forward	TGAAATCACCGCAGACGACA
	Reverse	ACACATTCCACCACCAGCTT
Notch1	Forward	CCAGCAGATGATCTTCCCGTAC
	Reverse	TAGACAATGGAGCCACGGATGT
Notch2	Forward	ATGTGGACGAGTGTCTGTTGC
	Reverse	GGAAGCATAGGCACAGTCATC
Notch3	Forward	TGCCAGAGTTCAGTGGTGG
	Reverse	CACAGGCAAATCGGCCATC
Notch4	Forward	CTCTTGCCACTCAATTTCCCT
	Reverse	TTGCAGAGTTGGGTATCCCTG
DLL1	Forward	AGGGTGTGATGACCAACATGGA
	Reverse	TATCGGATGCACTCATCGCAGTA
DLL4	Forward	TTCCAGGCAACCTTCTCCGA
	Reverse	ACTGCCGCTATTCTTGTCCC
Jagged1	Forward	CCTCGGGTCAGTTTGAGCTG
	Reverse	CCTTGAGGCACACTTTGAAGTA
Jagged2	Forward	CAATGACACCACTCCAGATGAG
	Reverse	GGCCAAAGAAGTCGTTGCG
Hes1	Forward	GCAGACATTCTGGAAATGACTGTGA
	Reverse	GAGTGCGCACCTCGGTGTTA
Hey1	Forward	CCGACGAGACCGAATCAATAAC
	Reverse	TCAGGTGATCCACAGTCATCTG
NRG1	Forward	GAGGTGAGAACACCCAAGTCA
	Reverse	TGGTCCCAGTCGTGGATGTAG
Impdh2	Forward	GATGGGGAGTCGATTGGTGG
	Reverse	GGTCTGTCCGGGCAATGATG
USP47	Forward	GATGTGATTCCCTTGGATTGCT
	Reverse	AACCCCATTGGTGTATCTTCTTC
MFF	Forward	ATGCCAGTGTGATAATGCAAGT
	Reverse	CTCGGCTCTCTTCGCTTTG
CD36	Forward	ATGGGCTGTGATCGGAACTG
	Reverse	GTCTTCTCAATAAGCATGTCTCC
LPL	Forward	GGGAGTTTGGCTCCAGAGTTT
	Reverse	TGTGTCTTCAGGGGTCCTTAG
Plin1	Forward	GGGACCTGTGAGTGCTTCC
	Reverse	GTATTGAAGAGCCGGGATCTTTT
Adipoq	Forward	TGTTCCTCTTAATCCTGCCCA
	Reverse	CCAACCTGCACAAGTTCCCTT
Fndc3b	Forward	GATGATGACCGACCAGATCCC
	Reverse	GCTCTGATGGTAAAGGTCTCACC
GAPDH	Forward	TGTGTCCGTCGTGGATCTGA
	Reverse	TTGCTGTTGAAGTCGCAGGAG


### Generation and Infection of the Recombinant Adenoviral Vector

The coding region of Notch1 was amplified by RT-PCR and cloned into the adenoviral vector pHBAd-EF1-MCS-GFP by *in vitro* recombination. The empty vector severed as an infection control. The recombinant plasmid was confirmed by DNA sequencing. The viral stocks were propagated in 293 cells. The cells were harvested 48 h after infection, and the concentrated virus was stored in aliquots at -80°C. For adenoviral infection, TSCs were grown to 50–70% confluence and infected with either the Ad-notch1 adenovirus or the control adenovirus. After 48–72 h, TSCs were harvested for different analysis.

### Small Interfering RNA (siRNA) Synthesis and Transfection

siRNA specifically targeting Notch1 and control siRNA (scrambled) were designed and synthesized by GenePharma (Suzhou, China). The siRNA sequences were 5′-GCUGGAUACAAGUGCAACUTT-3′ for Notch1 and 5′-UUCUCCGAACGUGUCACGUTT-3′ for scrambled control. Transfections were carried out using Lipofectamine^TM^ RNAiMAX Transfection Reagent (Invitrogen) following the manufacturer’s instructions. The transfected cells were harvested 48 or 72 h after transfection.

### Western Blot Analysis

Total proteins were extracted from cells using radioimmunoprecipitation assay (RIPA) lysis buffer (Beyotime Institute of Biotechnology, Shanghai, China), and protein concentrations were measured by the bicinchoninic acid (BCA) assay. Equal amounts of proteins were boiled at 100°C, separated by sodium dodecyl sulfate polyacrylamide gel electrophoresis (SDS-PAGE), and transferred onto polyvinylidene fluoride (PVDF) membranes (Millipore, Bedford, MA, United States). Afterward, the membranes were blocked with 5% (w/v) skim milk at room temperature and immunoblotted overnight at 4°C with rabbit polyclonal antibody against LaminB (Wanleibio, Shenyang, China) and rabbit monoclonal antibody against LC3, Beclin1, AKT, mammalian target of rapamycin (mTOR), Notch1, p62 (all from Abcam, Cambridge, MA, United States), p-AKT, p-mTOR (both from Cell Signaling Technology, Danvers, MA, United States) and β-tubulin (Beyotime Institute of Biotechnology). Then, appropriate horseradish peroxidase (HRP)-conjugated secondary antibodies were applied, and the protein bands were detected by the enhanced chemiluminescence (ECL) detection system. β-tubulin and LaminB were used as a cytoplasm and nuclear loading control, respectively.

### Flow Cytometry

When reaching 80–90% confluence, cells were harvested and fixed with paraformaldehyde at room temperature for 1 h. Then, 1 × 10^6^ cells (100 μl) were incubated with phycoerythrin (PE) labeled CD45 antibody (BioLegend, San Diego, CA, United States) at room temperature for 15 min in the dark. After permeabilization with 0.5% Triton X-100, cells were incubated with a rabbit monoclonal S1004A antibody (Abcam), followed by a corresponding Alexa Fluor^®^ conjugated secondary antibody. Finally, the samples were analyzed with a FACSCalibur flow cytometer (BD Biosciences, Sunnyvale, CA, United States).

### Oil Red O Staining

The lipid accumulation in differentiated cells were visualized by Oil Red O staining using a commercially available kit (Leagene Biotechnology, Beijing, China) as previously reported (Tan et al., 2017).

### Quantification of Intracellular Lipids

The intracellular lipids in rosiglitazone-induced TSCs were quantified by using AdipoRed^TM^ reagent (Lonza, Walkersville, MD, United States) as previously described (Tan et al., 2017). After rosiglitazone treatment, AdipoRed^TM^ reagent was added into each well and incubated at room temperature for 10 min. Fluorescence was measured on a plate reader with excitation set at 485 nm and emission at 572 nm.

### Immunofluorescence

Cells were grown on coverslips in 6-well plates, fixed in 4% paraformaldehyde at room temperature for 1 h, and permeabilized with 0.5% Triton X-100 in PBS for 20 min. After washing, unspecific binding sites were blocked with 5% FBS for 20 min at room temperature. Next, the coverslips were incubated overnight at 4°C with a rabbit monoclonal antibody against S100A4 (1:100 diluted, Abcam), followed by incubation with the fluorescein isothiocyanate (FITC)-labeled anti-rabbit IgG antibody. After stained the cell nuclei with 4′, 6-diamidino-2-phenylindole (DAPI), the coverslips were observed under a fluorescence microscopy.

### Statistical Analysis

Raw data are shown as mean ± standard deviation (SD). Statistical calculations were carried out using GraphPad Prism 5 (GraphPad Software, La Jolla, CA, United States). Differences among groups were analyzed by one-way analysis of variance (ANOVA) followed by the Bonferroni test for *post hoc* comparisons. A *P*-value less than 0.05 was considered to be statistically significant.

## Results

### Culture and Adipogenic Differentiation of TSCs

**FIGURE 1:**
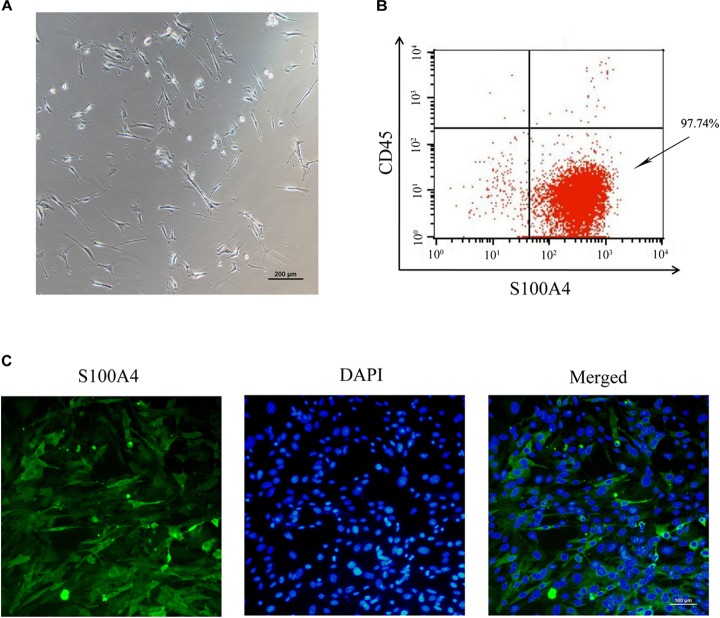
Culture and identification of TSCs. **(A)** The morphology of TSCs at passage 3 observed by optical microscope. **(B)** Flow cytometric analysis showed that nearly all the three-passage TSCs were CD45^-^S100A4^+^. **(C)** Immunofluorescence staining was imaged using anti-S100A4 (green), and the nuclei were counterstained with DAPI (blue).

**FIGURE 2:**
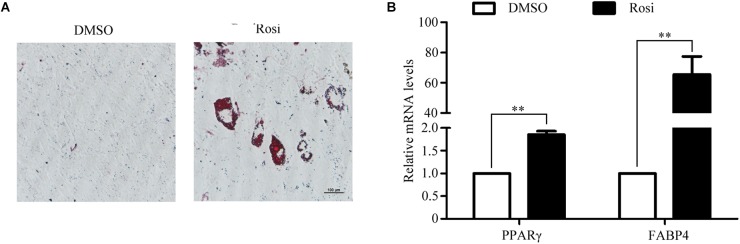
Adipogenic differentiation of TSCs. TSCs were treated with DMSO or rosiglitazone for 7 days to induce adipogenic differentiation. **(A)** Lipid accumulation was visualized by Oil Red O staining, and representative photographs of accumulated lipids are shown. **(B)** Real-time PCR was performed to detect FABP4 and PPARγ mRNA levels. The data were normalized to GAPDH. ^∗∗^*p* < 0.01. Rosi, rosiglitazone.

To establish an *in vitro* primary TSC culture model, postnatal thymi were subjected to enzymatic digestion, and thymocytes were depleted during passaging. Upon culture for three passages, TSCs displayed fibroblast-like phenotype (Figure 1A). Flow cytometry analysis showed that nearly all TSCs expressed S100A4, but exhibited negligible expression of CD45 (Figure 1B). Furthermore, immunostaining showed that all TSCs were also positive for S100A4, a fibroblast marker (Figure 1C). To induce adipogenic differentiation, TSCs were treated with PPARγ ligand rosiglitazone for 7 days, and Oil red O staining was performed to visualize lipid accumulation. Compared with DMSO-treated cells, intracellular lipid droplets were observed in TSCs treated with rosiglitazone (Figure 2A). In addition, real-time PCR analysis showed that the mRNA levels of adipogenic markers, PPARγ and fatty acid binding protein 4 (FABP4), were remarkably increased after rosiglitazone treatment (Figure 2B; *P* < 0.01). Therefore, we successfully cultured primary TSCs, which offer us a useful cellular model to investigate the mechanisms underlying thymic adipogenesis.

### Identification of Differentially Expressed Genes During Adipogenic Transformation in TSCs by RNA-Seq

**FIGURE 3:**
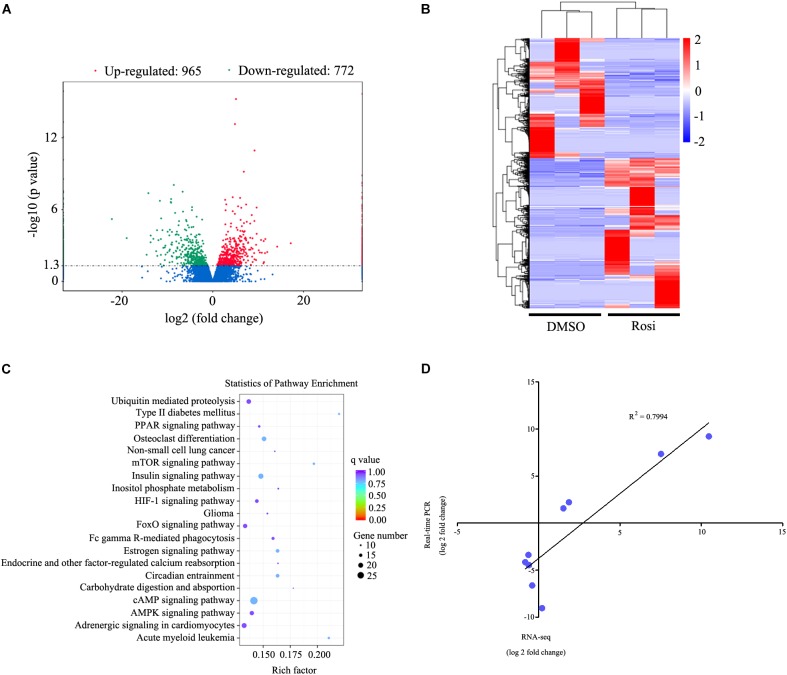
RNA-seq analysis of genes differed significantly in rosiglitazone-treated cells compared with DMSO-treated cells. **(A)** Volcano plot of differentially expressed genes between rosiglitazone-treated cells compared with DMSO-treated cells. Fold changes (X axis) are plotted against statistical significance (Y axis) for each gene. Genes up-regulated with a fold change > 2 and *p* < 0.05 are indicated in red, and those down-regulated with a fold change > 2 and *p* < 0.05 are depicted in green. **(B)** Cluster analysis of the genes that were up-regulated and down-regulated in rosiglitazone-treated cells compared with DMSO-treated cells (*p* < 0.05, fold change > 2). A dendrogram of the cluster correlation is present on the right. Pseudocolors show differential expression (red indicates gene levels greater than the median; white indicates gene levels equal to the median; blue indicates gene levels below the median). **(C)** Pathway analysis of differentially expressed genes based on KEGG database. The color and size of each circle was based on the significance and the number of genes contained in the pathway, respectively. **(D)** Comparison of RNA-seq and real-time PCR expression measurements for the selected genes. Graph shows log of fold changes for RNA-seq data and log of fold changes for real-time PCR data. Rosi, rosiglitazone.

In order to identify key regulators in thymic adipogenesis, RNA-seq analysis was carried out in DMSO- and rosiglitazone-treated TSCs. As a result, we identified a total of 1737 genes differed significantly at *P* < 0.05 with a fold change > 2, among which 965 genes were up-regulated and 772 genes were down-regulated in rosiglitazone-treated cells compared with DMSO-treated cells. The complete list of these genes is shown in Supplementary Table S1. The differentially expressed genes are also represented in a volcano plot (Figure 3A). Unsupervised hierarchical clustering analysis revealed that DMSO-treated cells are distinctly different from rosiglitazone-treated cells (Figure 3B). In order to identify the potential functions of these differentially expressed genes, we performed gene ontology (GO) enrichment analysis and found that the GO terms were enriched in metabolic process, intracellular, and protein binding (Supplementary Table S2). KEGG analysis was carried out to assess the involved pathways of the differentially expressed genes. The results showed that a number of pathways, including ubiquitin mediated proteolysis, PPAR signaling pathway, and mTOR signaling pathway were predominantly over-represented (Figure 3C; *P* < 0.05). To validate the results of RNA-seq analysis, real-time PCR was conducted to detect a number of candidate genes (Table 1). Fold-change differences detected by real-time PCR were not as pronounced as those observed in RNA-seq analysis. However, when the log of the fold change in gene expression as determined by real-time PCR was plotted against the log of the fold change as determined by RNA-seq analysis, there was a clear correlation between the two methods (*R*^2^= 0.7994; Figure 3D). These data indicate that the results observed by real-time PCR were consistent with those of RNA-seq analysis.

### Overexpression of Notch1 Attenuated and Inhibition of Notch1 Promoted Rosiglitazone-Induced Adipogenic Differentiation in TSCs

**FIGURE 4:**
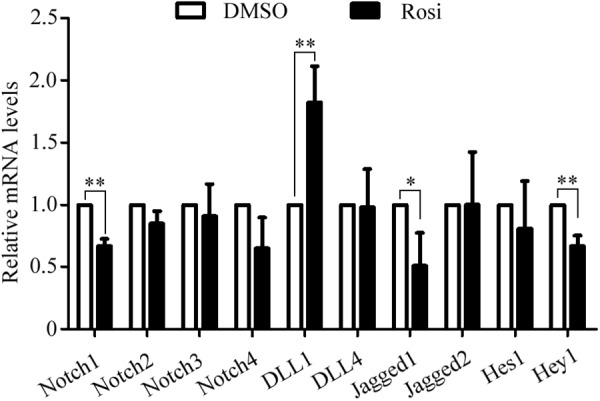
The mRNA levels of multiple members in the Notch pathway analyzed by Real-time PCR analysis. The data were normalized to GAPDH, and the graph represents the results of three independent experiments. ^∗∗^*p* < 0.01; ^∗^*p* < 0.05. Rosi, rosiglitazone.

**FIGURE 5:**
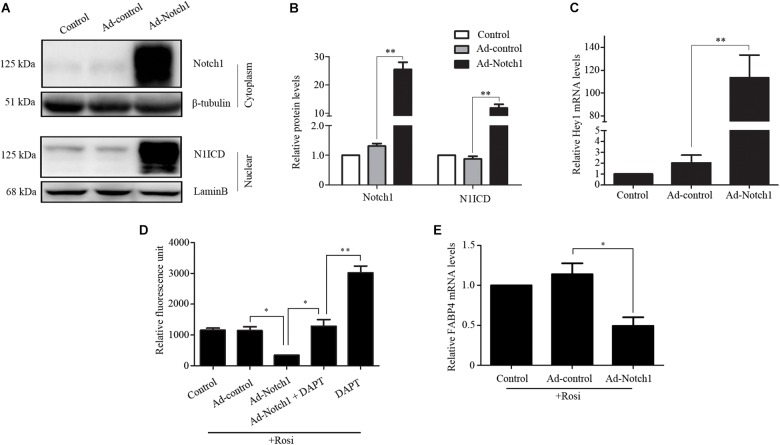
Notch1 regulated rosiglitazone-induced adipogenic differentiation in TSCs. **(A)** Western blot analysis of cytoplasm Notch1 and nuclear N1ICD expression in adenoviral vector infected TSCs. Representative blots of three independent experiments are shown, and the protein size is expressed in kDa. **(B)** Quantification of Notch1 protein levels from three separate experiments. **(C)** Real-time PCR analysis of Hey1 mRNA levels in adenoviral vector infected TSCs. Adenoviral vector infected TSCs were treated with rosiglitazone for 7 days to induce adipogenic differentiation in the presence or absence of DAPT. Intracellular triglyceride content was determined with AdipoRed Assay Reagent **(D)**, and FABP4 mRNA levels were detected by real-time PCR analysis **(E)**. ^∗∗^*p* < 0.01; ^∗^*p* < 0.05. Rosi, rosiglitazone.

**FIGURE 6:**
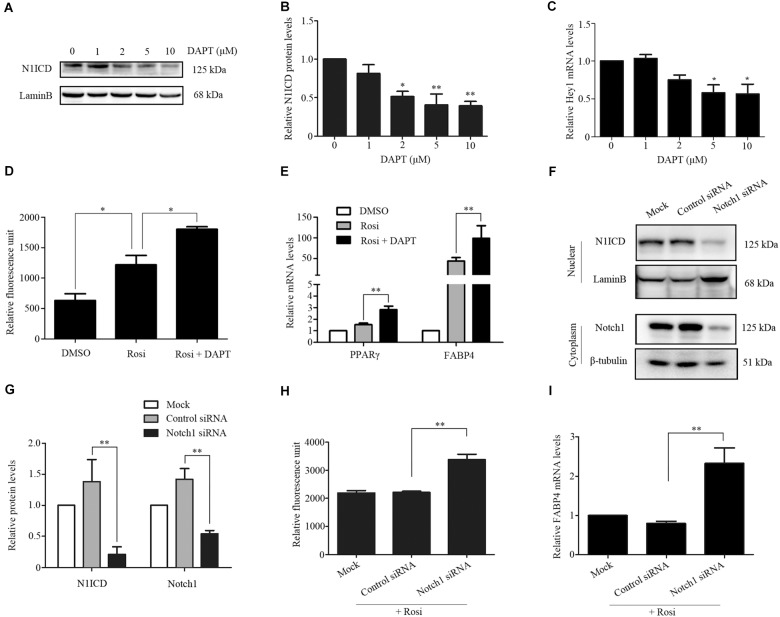
TSCs were treated with various concentrations of DAPT, and Western blot analysis and real-time PCR was performed to measure nuclear N1ICD protein levels **(A,B)** and Hey1 mRNA levels **(C)**, respectively. TSCs were treated with rosiglitazone for 7 days to induce adipogenic differentiation in the presence or absence of DAPT. Intracellular triglyceride content was assessed with AdipoRed Assay Reagent **(D)**, and FABP4 and PPARγ mRNA levels were detected by real-time PCR analysis **(E)**. Specific gene silencing of Notch1 appeared in siRNA-transfected TSCs by Western blot analysis **(F,G)**. siRNA-transfected TSCs were treated with rosiglitazone for 7 days to induce adipogenic differentiation. Intracellular triglyceride content was determined with AdipoRed Assay Reagent **(H)**, and FABP4 mRNA levels were detected by real-time PCR analysis **(I)**. ^∗∗^*p* < 0.01; ^∗^*p* < 0.05. Rosi, rosiglitazone.

We note that several members of the Notch pathway were significantly altered in RNA-seq analysis. Therefore, real-time PCR was carried out to evaluate the expression of multiple members in the Notch pathway. We found that Notch1, Jagged1 and Hey1 were significantly down-regulated, while DLL1 was dramatically up-regulated in rosiglitazone-treated TSCs compared to control TSCs (Figure 4), suggesting a potential role of the Notch pathway in thymic adipogenesis. Next, the adenoviral vector carrying Notch1 gene was constructed and transduced into TSCs. Substantial increases in Notch1 protein levels, N1ICD nuclear translocation (Figures 5A,B; *P* < 0.01), and Hey1 mRNA levels (Figure 5C; *P* < 0.01) were observed in TSCs infected with adenoviral vector carrying Notch1 gene in comparison to control cells, suggesting activation of Notch pathway. Moreover, the effects of Notch1 on adipogenic differentiation were investigated by measuring the intracellular triglyceride content with a commercially available AdipoRed Assay Reagent. We found that overexpression of Notch1 inhibited rosiglitazone-induced increases in intracellular triglyceride content (Figure 5D) and FABP4 mRNA levels (Figure 5E) in TSCs. In contrast, DAPT, an inhibitor of Notch signaling, potently attenuated the inhibitory effects of Notch1 overexpression on adipogenic differentiation (Figure 5D). Furthermore, DAPT down-regulated the nuclear expression of N1ICD protein (Figures 6A,B) and Hey1 mRNA levels (Figure 6C) in a dose-dependent manner. DAPT also substantially promoted rosiglitazone-induced increases in intracellular triglyceride content (Figure 6D) and PPARγ and FABP4 mRNA levels (Figure 6E). To further characterize the role of Notch1 in adipogenic differentiation of TSCs, we employed an RNA interference approach to knockdown Notch1 expression in TSCs and found that both cytoplasm Notch1 and nuclear N1ICD protein levels in cells transfected with specific siRNA for Notch1 were significantly reduced compared with those transfected with control siRNA (Figures 6F,G). Similar to DAPT, knockdown of Notch1 also dramatically promoted rosiglitazone-induced increases in intracellular triglyceride content (Figure 6H) and FABP4 mRNA levels (Figure 6I). Taken together, these findings demonstrate that Notch1 negatively regulates rosiglitazone-induced adipogenic differentiation in TSCs.

### DAPT, an Inhibitor of Notch Signaling, Induced Autophagy and Adipogenic Differentiation in TSCs via Inhibition of AKT/mTOR Pathway

**FIGURE 7:**
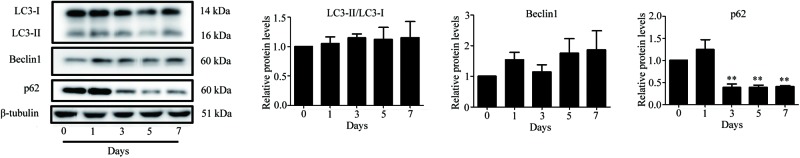
Increased autophagic activity during adipogenic differentiation of TSCs. TSCs were treated with rosiglitazone as indicated intervals. The expression levels of several autophagy markers, including LC3, Beclin1, and p62 were determined by Western blot analysis. Densitometric quantification data are expressed as the intensity ratio of target proteins to β-tubulin. ^∗∗^*p* < 0.01.

**FIGURE 8:**
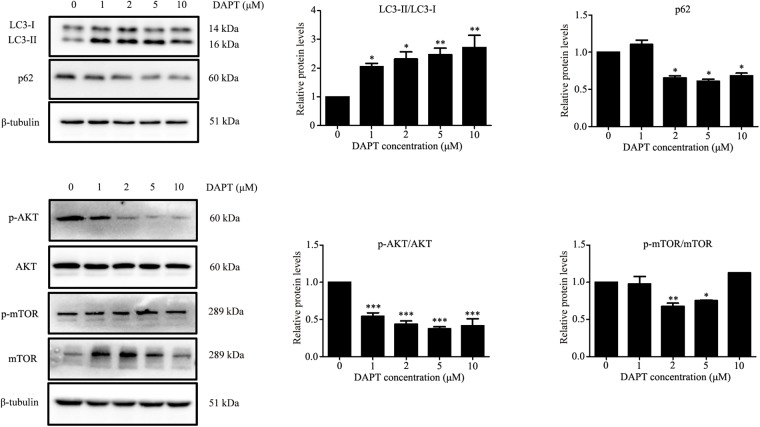
DAPT, an inhibitor of Notch signaling, induced autophagy in TSCs. TSCs were treated with various concentrations of DAPT for 48 h. Western blot analysis was conducted to determine the levels of LC3, p62, AKT, p-AKT, mTOR, and p-mTOR. Densitometric values were normalized by β-tubulin. ^∗∗∗^*p* < 0.001; ^∗∗^*p* < 0.01; ^∗^*p* < 0.05.

**FIGURE 9:**
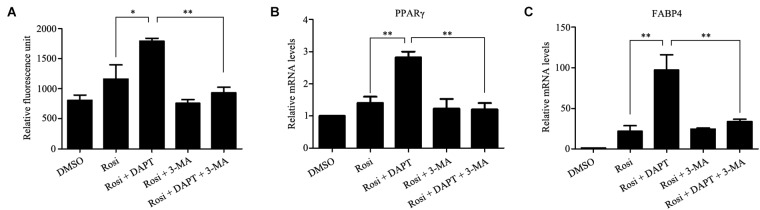
The pro-adipogenic effects of DAPT in TSCs are associated with the activation of autophagy. TSCs were incubated with autophagy inhibitor 3-MA (5 mM) in the absence or presence of DAPT during adipogenesis. Adipogenesis was determined by measuring intracellular triglyceride content **(A)** and mRNA levels of PPARγ **(B)**, and FABP4 **(C)**. ^∗∗^*p* < 0.01; ^∗^*p* < 0.05; Rosi, rosiglitazone; 3-MA, 3-methyladenine.

**FIGURE 10:**
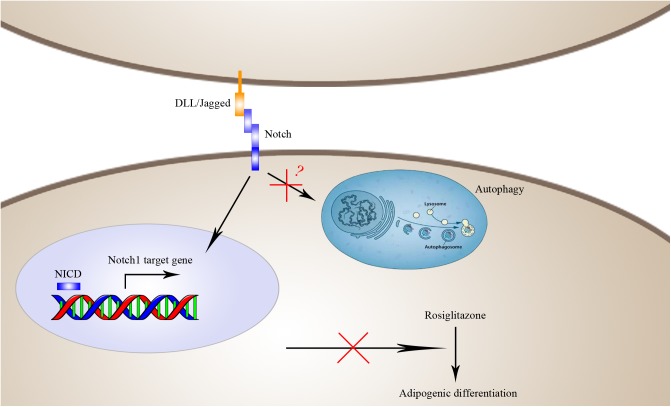
Schematic diagram of the mechanisms by which Notch1 inhibits rosiglitazone-induced adipogenic differentiation in TSCs. Activation of Notch1 induces nuclear expression NICD to transcriptionally up-regulate the target gene Hey1 and thereby inhibits rosiglitazone-induced adipogenic differentiation in TSCs.

Autophagy is known to be a degradation process of intracellular proteins and organelles during adipogenesis (Goldman et al., 2011; Zhang et al., 2012; Ahmed et al., 2018). To investigate the role of autophagy in adipogenic differentiation in TSCs, the expression levels of several autophagy markers, including LC3, Beclin1, and p62 during adipogenesis were determined by Western blot analysis. As illustrated in Figure 7, p62 was down-regulated from day 3 afterward, suggesting activation of autophagy during adipogenesis. However, the levels of LC3-II/I and Beclin1 remained unchanged during adipogenesis (Figure 7). To evaluate the effects of Notch signaling on autophagy, TSCs were treated with various concentrations of DAPT for 48 h. We found that DAPT dose-dependently increased the ratio of LC3-II/LC3-I, but reduced the protein levels of p62 in TSCs (Figure 8). Meanwhile, DAPT also decreased the levels p-AKT and p-mTOR in a dose-dependent fashion (Figure 8). To test whether DAPT-induced adipogenesis is dependent on autophagy activation, TSCs were incubated with autophagy inhibitor 3-MA (5 mM) in the absence or presence of DAPT during adipogenesis. Adipogenesis was determined by measuring intracellular triglyceride content and mRNA levels of PPARγ and FABP4. We found that 3-MA abolished the pro-adipogenic effects of DAPT in TSCs (Figures 9A–C), suggesting that the pro-adipogenic effects of DAPT in TSCs are associated with the activation of autophagy. Collectively, our results reveal that Notch1 inhibits rosiglitazone-induced adipogenic differentiation in TSCs (Figure 10).

## Discussion

In the present study, we successfully cultured primary TSCs and performed a comprehensive analysis of differentially expressed genes during adipogenesis of TSCs by using RNA-seq method. As a result, we identified a total of 1737 differentially expressed genes, among which 965 genes were up-regulated and 772 genes were down-regulated in rosiglitazone-treated cells compared with DMSO-treated cells. GO enrichment analysis showed that the GO terms were enriched in metabolic process, intracellular, and protein binding. KEGG analysis showed that a number of pathways, including ubiquitin mediated proteolysis, PPAR signaling pathway, and mTOR signaling pathway were predominantly over-represented. Our further experiments demonstrated that overexpression of Notch1 suppressed, while the Notch signaling inhibitor DAPT promoted adipogenic differentiation in TSCs, and the pro-adipogenic effects of DAPT were associated with the activation of autophagy. Taken together, our results suggest that Notch1 is a key regulator in thymic adipogenesis and may serve as a potential target to hinder thymic adiposity in age-related thymic involution.

The thymus is constituted by a myriad of cell types, such as thymocyte, macrophages, fibroblasts, dendritic cells, and epithelial cells (Xing and Hogquist, 2014; Tan J.X. et al., 2016). In the present study, postnatal thymi were subjected to enzymatic digestion, and suspension cells were cultured. Upon culture, approximately 97.74% cells were CD45^-^S100A4^+^ and exhibited fibroblast-like phenotype. This is largely due to the fact that thymocytes are removed by refreshing the medium, and fibroblasts proliferate at a faster rate than other cells. Our results are consistent with the findings by Yang et al. (2009). This culture system offers us a useful cellular model to investigate the mechanisms underlying thymic adipogenesis.

Age-related thymic involution is accompanied by loss of thymic epithelial cell (TECs) and increase in fibroblasts (Gray et al., 2007; Aw et al., 2008). Several lines of evidence demonstrate that fibroblasts can be readily induced to develop into adipocytes and represent a cellular source of thymic adipogenesis (Youm et al., 2009; Tan J. et al., 2016; Sheridan et al., 2017). The nuclear receptor PPARγ has been shown to maintain insulin sensitivity and stimulate adipogenesis (Oger et al., 2014). PPARγ plays a key role in white adipose tissue differentiation, and forced PPARγ expression in nonadipogenic cells leads to their adipocyte differentiation (Evans et al., 2004; Rosen and MacDougald, 2006). Furthermore, Yang et al. (2009) have reported high PPARγ protein levels in aging thymus, suggesting PPARγ-driven adipogenesis of thymic fibroblasts. Rosiglitazone, a PPARγ ligand, has been commonly used to induce transdifferentiation of multipotential cells into adipocytes, such as proadipocytes (Kim et al., 2016), bone marrow-derived mesenchymal stem cells (Lu et al., 2016), and embryonic stem cells (Xiong et al., 2005). We and other have also used rosiglitazone to induce adipocyte differentiation of OP9-DL1 cells (Tan et al., 2017) and primary TSCs (Yang et al., 2009). In the present study, we performed a comprehensive analysis of differentially expressed genes during adipogenesis of TSCs by using RNA-seq method and identified a total of 1737 differentially expressed genes. GO enrichment analysis showed that the GO terms were enriched in metabolic process, intracellular, and protein binding. KEGG analysis showed that a number of pathways, including ubiquitin mediated proteolysis, PPAR signaling pathway, and mTOR signaling pathway were predominantly over-represented. However, further investigations are needed to uncover the functions of these altered genes in adipocyte differentiation of TSCs.

The Notch signaling pathway is a highly conserved signaling pathway that regulates cell proliferation, cell fate, differentiation, and cell death (Yamamoto et al., 2014). Currently, the function of Notch signaling pathway in adipocyte differentiation remains controversial (Shan et al., 2017). Activation of Notch signaling inhibits the differentiation of 3T3-L1 cells by regulating the target gene Hes1 (Ross et al., 2004). Mechanically, Hes1 can directly repress expression of C/EBPα and PPARγ (Ross et al., 2006). Song et al have documented that inhibition of Notch signaling promotes adipogenic differentiation of mesenchymal stem cells (Song et al., 2015). Nonetheless, inhibition of Notch signaling by soluble Jagged1 blocks the differentiation of 3T3-L1 preadipocytes (Ross et al., 2004). In the present study, we note that several members of Notch pathways were significantly altered in RNA-seq analysis. Therefore, real-time PCR was carried out to evaluate the expression of multiple members in the Notch pathway. We found that Notch1, Jagged1 and Hey1 were significantly down-regulated, while DLL1 was dramatically up-regulated in rosiglitazone-treated TSCs compared to control TSCs, suggesting a potential role of the Notch pathway in thymic adipogenesis. Our further experiments showed that overexpression of Notch1 inhibited, and DAPT, an inhibitor of Notch signaling, and knockdown of Notch1 promoted rosiglitazone-induced adipogenic differentiation in TSCs. Consequently, our data support a negative role of Notch signaling in adipogenic differentiation of TSCs. However, Aw and co-workers found increased expression of Notch1 in the thymi of older mice using immunohistochemistry (Aw et al., 2009). This conflict could be explained by the fact that upregulation of Notch1 with increasing age, amongst the TEC populations, is mostly confined to those within the cortex, and other cell types, including thymocytes may contribute to the rise in Notch1 expression in older mice (Aw et al., 2009). Thus, it is essential to analyze the expression of Notch1 in specific thymic cell populations.

Recent evidence suggests a pivotal role of autophagy in adipocyte differentiation (Tao et al., 2017), and increased autophagy in adipose tissues has been observed in obese humans and animals (Kovsan et al., 2011; Jansen et al., 2012; Nunez et al., 2013). In this study, we consistently found increased autophagy in adipogenic differentiation of TSCs, as evidence by reduced expression of p62. To further clarify the mechanisms by which Notch signaling pathway mediates adipogenic differentiation of TSCs, the effects of DAPT on LC3, p62, and AKT/mTOR were determined by Western blot analysis. We found that DAPT increased the ratio of LC3-II/LC3-I, but reduced the protein levels of p62, p-AKT and p-mTOR in TSCs, suggesting increased autophagy. Additionally, we demonstrated that 3-MA, an autophagy inhibitor abolished the pro-adipogenic effects of DAPT in TSCs, suggesting that the pro-adipogenic effects of DAPT in TSCs were associated with the activation of autophagy. These results are largely in agreement with results by Song et al in mesenchymal stem cells (Song et al., 2015). Moreover, the autophagic process is essential for the presentation of self-Ags in the thymus to shape the T-cell repertoires (Kasai et al., 2009), and TECs possess a high constitutive level of autophagy, which is decreased with advancing of age (Uddin et al., 2012). However, the autophagic activity in thymic fibroblasts at different ages needs to be further investigated.

## Conclusion

In conclusion, our RNA-seq analysis identified a number of differentially expressed genes and associated signaling pathways in the adipocyte differentiation of primary TSCs. Moreover, we demonstrated that Notch1 inhibits rosiglitazone-induced adipogenic differentiation in TSCs, and increased autophagy was observed in rosiglitazone-induced adipogenic differentiation in TSCs. Our findings not only offer the basis for identification of key mediators in thymic adipogenesis, but also uncover a negative role of Notch1 in thymic adiposity. Activation of Notch1 pathway may be an effective strategy to inhibit thymic adiposity in age-related thymic involution.

## Author Contributions

YW, JT, and XZ conceived and designed the experiments. YW, JT, HD, and XL performed the experiments. HD and SilW analyzed the data. YW, JT, SimW, and ZY contributed reagents, materials, analysis tools. YW and XZ wrote the paper.

## Conflict of Interest Statement

The authors declare that the research was conducted in the absence of any commercial or financial relationships that could be construed as a potential conflict of interest.

## Supplementary Material

TABLE S1 The complete list of differentially expressed genes.

TABLE S2 Enriched GO terms of differentially expressed genes.

## References

[B1] Ahmed M. Hwang J. S. Lai T. H. Zada S. Nguyen H. Q. Pham T. M. (2018). Co-Expression network analysis of AMPK and autophagy gene products during adipocyte differentiation. *Int. J. Mol. Sci.* 19:E1808. 10.3390/ijms19061808 29921805 PMC6032425

[B2] Aw D. Palmer D. B. (2011). The origin and implication of thymic involution. *Aging Dis.* 2 437–443. 22396892 PMC3295077

[B3] Aw D. Silva A. B. Maddick M. von Zglinicki T. Palmer D. B. (2008). Architectural changes in the thymus of aging mice. *Aging Cell* 7 158–167. 10.1111/j.1474-9726.2007.00365.x 18241323

[B4] Aw D. Taylor-Brown F. Cooper K. Palmer D. B. (2009). Phenotypical and morphological changes in the thymic microenvironment from ageing mice. *Biogerontology* 10 311–322. 10.1007/s10522-008-9182-2 18931936

[B5] Chaudhry M. S. Velardi E. Dudakov J. A. van den Brink M. R. (2016). Thymus: the next (re)generation. *Immunol. Rev.* 271 56–71. 10.1111/imr.12418 27088907 PMC4837659

[B6] Ernszt D. Banfai K. Kellermayer Z. Pap A. Lord J. M. Pongracz J. E. (2017). PPARgamma deficiency counteracts thymic senescence. *Front. Immunol.* 8:1515. 10.3389/fimmu.2017.01515 29163553 PMC5681731

[B7] Evans R. M. Barish G. D. Wang Y. X. (2004). PPARs and the complex journey to obesity. *Nat. Med.* 10 355–361. 10.1038/nm1025 15057233

[B8] Flores K. G. Li J. Sempowski G. D. Haynes B. F. Hale L. P. (1999). Analysis of the human thymic perivascular space during aging. *J. Clin. Invest.* 104 1031–1039. 10.1172/JCI7558 10525041 PMC408578

[B9] Goldman S. J. Zhang Y. Jin S. (2011). Autophagic degradation of mitochondria in white adipose tissue differentiation. *Antioxid. Redox. Signal.* 14 1971–1978. 10.1089/ars.2010.3777 21126221 PMC3078505

[B10] Gray D. H. Tull D. Ueno T. Seach N. Classon B. J. Chidgey A. (2007). A unique thymic fibroblast population revealed by the monoclonal antibody MTS-15. *J. Immunol.* 178 4956–4965. 10.4049/jimmunol.178.8.4956 17404277

[B11] Gui J. Mustachio L. M. Su D. M. Craig R. W. (2012). Thymus size and age-related thymic involution: early programming, sexual dimorphism, progenitors and stroma. *Aging Dis.* 3 280–290. 22724086 PMC3375084

[B12] Jansen H. J. van Essen P. Koenen T. Joosten L. A. Netea M. G. Tack C. J. (2012). Autophagy activity is up-regulated in adipose tissue of obese individuals and modulates proinflammatory cytokine expression. *Endocrinology* 153 5866–5874. 10.1210/en.2012-1625 23117929

[B13] Kasai M. Tanida I. Ueno T. Kominami E. Seki S. Ikeda T. (2009). Autophagic compartments gain access to the MHC class II compartments in thymic epithelium. *J. Immunol.* 183 7278–7285. 10.4049/jimmunol.0804087 19915056

[B14] Kim J. Lee Y. J. Kim J. M. Lee S. Y. Bae M. A. Ahn J. H. (2016). PPARgamma agonists induce adipocyte differentiation by modulating the expression of Lipin-1, which acts as a PPARgamma phosphatase. *Int. J. Biochem. Cell Biol.* 81(Pt A), 57–66. 10.1016/j.biocel.2016.10.018 27780754

[B15] Kovsan J. Bluher M. Tarnovscki T. Kloting N. Kirshtein B. Madar L. (2011). Altered autophagy in human adipose tissues in obesity. *J. Clin. Endocrinol. Metab.* 96 E268–E277. 10.1210/jc.2010-1681 21047928

[B16] Lu W. Wang W. Wang S. Feng Y. Liu K. (2016). Rosiglitazone promotes bone marrow adipogenesis to impair myelopoiesis under stress. *PLoS One* 11:e0149543. 10.1371/journal.pone.0149543 26895498 PMC4760757

[B17] McGettigan P. A. (2013). Transcriptomics in the RNA-seq era. *Curr. Opin. Chem. Biol.* 17 4–11. 10.1016/j.cbpa.2012.12.008 23290152

[B18] Nunez C. E. Rodrigues V. S. Gomes F. S. Moura R. F. Victorio S. C. Bombassaro B. (2013). Defective regulation of adipose tissue autophagy in obesity. *Int. J. Obes.* 37 1473–1480. 10.1038/ijo.2013.27 23478428

[B19] Oger F. Dubois-Chevalier J. Gheeraert C. Avner S. Durand E. Froguel P. (2014). Peroxisome proliferator-activated receptor gamma regulates genes involved in insulin/insulin-like growth factor signaling and lipid metabolism during adipogenesis through functionally distinct enhancer classes. *J. Biol. Chem.* 289 708–722. 10.1074/jbc.M113.526996 24288131 PMC3887199

[B20] Rosen E. D. MacDougald O. A. (2006). Adipocyte differentiation from the inside out. *Nat. Rev. Mol. Cell Biol.* 7 885–896. 10.1038/nrm2066 17139329

[B21] Ross D. A. Hannenhalli S. Tobias J. W. Cooch N. Shiekhattar R. Kadesch T. (2006). Functional analysis of Hes-1 in preadipocytes. *Mol. Endocrinol.* 20 698–705. 10.1210/me.2005-0325 16282371

[B22] Ross D. A. Rao P. K. Kadesch T. (2004). Dual roles for the Notch target gene Hes-1 in the differentiation of 3T3-L1 preadipocytes. *Mol. Cell. Biol.* 24 3505–3513. 10.1128/MCB.24.8.3505-3513.2004 15060169 PMC381674

[B23] Shan T. Liu J. Wu W. Xu Z. Wang Y. (2017). Roles of notch signaling in adipocyte progenitor cells and mature adipocytes. *J. Cell. Physiol.* 232 1258–1261. 10.1002/jcp.25697 27869309

[B24] Sheridan J. M. Keown A. Policheni A. Roesley S. N. A. Rivlin N. Kadouri N. (2017). Thymospheres are formed by mesenchymal cells with the potential to generate adipocytes, but not epithelial cells. *Cell Rep.* 21 934–942. 10.1016/j.celrep.2017.09.090 29069601

[B25] Song B. Q. Chi Y. Li X. Du W. J. Han Z. B. Tian J. J. (2015). Inhibition of notch signaling promotes the adipogenic differentiation of mesenchymal stem cells through autophagy activation and PTEN-PI3K/AKT/mTOR Pathway. *Cell Physiol. Biochem.* 36 1991–2002. 10.1159/000430167 26202359

[B26] Tan J. Wang Y. Wang S. Zhang N. Wu S. Yuan Z. (2017). Untargeted metabolomics analysis of adipogenic transformation in OP9-DL1 cells using liquid chromatography-mass spectrometry: implications for thymic adipogenesis. *Cell Biol. Int.* 41 447–456. 10.1002/cbin.10740 28185342

[B27] Tan J. Wang Y. Zhang N. Zhu X. (2016). Induction of epithelial to mesenchymal transition (EMT) and inhibition on adipogenesis: two different sides of the same coin? Feasible roles and mechanisms of transforming growth factor beta1 (TGF-beta1) in age-related thymic involution. *Cell Biol. Int.* 40 842–846. 10.1002/cbin.10625 27189906

[B28] Tan J. X. Wang Y. J. Zhu X. K. (2016). [Progresses in therapeutic strategies for thymic rejuvenation]. *Sheng Li Xue Bao* 68 75–86. 26915325

[B29] Tao Z. Liu L. Zheng L. D. Cheng Z. (2017). Autophagy in adipocyte differentiation. *Methods Mol. Biol.* 1854 45–53. 10.1007/7651_2017_65 28815517

[B30] Uddin M. N. Nishio N. Ito S. Suzuki H. Isobe K. (2012). Autophagic activity in thymus and liver during aging. *Age* 34 75–85. 10.1007/s11357-011-9221-9 21387084 PMC3260356

[B31] Ventevogel M. S. Sempowski G. D. (2013). Thymic rejuvenation and aging. *Curr. Opin. Immunol.* 25 516–522. 10.1016/j.coi.2013.06.002 23831111 PMC3775968

[B32] Wang Z. Gerstein M. Snyder M. (2009). RNA-Seq: a revolutionary tool for transcriptomics. *Nat. Rev. Genet.* 10 57–63. 10.1038/nrg2484 19015660 PMC2949280

[B33] Xing Y. Hogquist K. A. (2014). Isolation, identification, and purification of murine thymic epithelial cells. *J. Vis. Exp.* 90:e51780. 10.3791/51780 25145384 PMC4374365

[B34] Xiong C. Xie C. Q. Zhang L. Zhang J. Xu K. Fu M. (2005). Derivation of adipocytes from human embryonic stem cells. *Stem Cells Dev.* 14 671–675. 10.1089/scd.2005.14.671 16433622

[B35] Yamamoto S. Schulze K. L. Bellen H. J. (2014). Introduction to Notch signaling. *Methods Mol. Biol.* 1187 1–14. 10.1007/978-1-4939-1139-4_1 25053477

[B36] Yang H. Youm Y. H. Dixit V. D. (2009). Inhibition of thymic adipogenesis by caloric restriction is coupled with reduction in age-related thymic involution. *J. Immunol.* 183 3040–3052. 10.4049/jimmunol.0900562 19648267 PMC2731487

[B37] Youm Y. H. Yang H. Amin R. Smith S. R. Leff T. Dixit V. D. (2010). Thiazolidinedione treatment and constitutive-PPARgamma activation induces ectopic adipogenesis and promotes age-related thymic involution. *Aging Cell* 9 478–489. 10.1111/j.1474-9726.2010.00574.x 20374200 PMC2910128

[B38] Youm Y. H. Yang H. Sun Y. Smith R. G. Manley N. R. Vandanmagsar B. (2009). Deficient ghrelin receptor-mediated signaling compromises thymic stromal cell microenvironment by accelerating thymic adiposity. *J. Biol. Chem.* 284 7068–7077. 10.1074/jbc.M808302200 19054770 PMC2652314

[B39] Zhang Y. Zeng X. Jin S. (2012). Autophagy in adipose tissue biology. *Pharmacol. Res.* 66 505–512. 10.1016/j.phrs.2012.09.004 23017672

[B40] Zhao S. Fung-Leung W. P. Bittner A. Ngo K. Liu X. (2014). Comparison of RNA-Seq and microarray in transcriptome profiling of activated T cells. *PLoS One* 9:e78644. 10.1371/journal.pone.0078644 24454679 PMC3894192

